# Levetiracetam may be an unsuitable choice for patients with *PRRT2*-associated self-limited infantile epilepsy

**DOI:** 10.1186/s12887-023-04212-w

**Published:** 2023-10-25

**Authors:** Yang Tian, Zhen Shi, Jiahao Cai, Chi Hou, Xiuying Wang, Haixia Zhu, Binwei Peng, Kaili Shi, Xiaojing Li, Sitang Gong, Wen-Xiong Chen

**Affiliations:** 1 The First Affiliated Hospital of Jinan University, Jinan University, Guangzhou, China; 2 Department of Neurology, Guangzhou Women and Children’s Medical Center, Guangzhou Medical University, Guangdong Provincial Clinical Research Center for Child Health, Guangzhou, 510623 China; 3 Department of Neurology, Guangzhou Women and Children’s Medical Center, Guangzhou Medical University, 9# Jin Sui Road, Guangzhou, 510623 China; 4 Department of Pediartic, Guangzhou Women and Children’s Medical Center, Guangzhou Medical University, 9# Jin Sui Road, Guangzhou, 510623 China

**Keywords:** Self-limited infantile epilepsy, Levetiracetam, Antiseizure medication, *PRRT2*, Microdeletion, Treatment

## Abstract

**Introduction:**

Self-limited infantile epilepsy (SeLIE) is a benign epilepsy. Previous studies have shown that monotherapy with most antiseizure medications can effectively relieve seizures in patients with SeLIE, but the efficacy of levetiracetam has not been investigated.

**Objective:**

This study aimed to investigate the efficacy of levetiracetam in the treatment of SeLIE patients with *PRRT2* mutations.

**Methods:**

The clinical data of 39 SeLIE patients (21 males and 18 females, aged 4.79 ± 1.60 months) with pathogenic variants in *PRRT2* or 16p11.2 microdeletion were retrospectively analyzed. Based on the use of initial antiseizure medication (ASM), the patients were classified into two groups: Levetiracetam group (LEG) and Other ASMs group (OAG). The difference of efficacy between the two groups was compared.

**Results:**

Among the 39 SeLIE patients, 16 were LEG (10 males and 6 females, aged 5.25 ± 2.07 months), with whom two obtained a seizure-free status (12.50%) and 14 ineffective or even deteriorated (87.50%). Among the 14 ineffective or deteriorated cases, 13 were seizure-controlled after replacing levetiracetam with other ASMs including topiramate, oxcarbazepine, lamotrigine, and valproate, and the remaining one finally achieved remission at age 3. Of the 39 patients, 23 were OAG (11 males and 12 females; aged 4.48 ± 1.12 months), of whom 22 achieved seizure remission, except for one patient who was ineffective with topiramate initially and relieved by oxcarbazepine instead. Although there were no significant differences in gender and age of onset between the two groups, the effective rate was significantly different (12.50% in LEG vs. 95.65% in OAG) (*P* < 0.01).

**Conclusion:**

The findings showed that patients with SeLIE caused by the *PRRT2* mutations did not benefit from the use of levetiracetam, but could benefit from other ASMs.

**Supplementary Information:**

The online version contains supplementary material available at 10.1186/s12887-023-04212-w.

## Introduction

Self-limited infantile epilepsy (SeLIE) has been established as a distinct epilepsy syndrome characterized by focal seizures occurring in clusters of infantile-onset, with normal interictal electroencephalogram (EEG) and neuroimaging. Approximately 40% of the children with SeLIE are reported to have a positive family history [1], also known as self-limited familial infantile epilepsy (SeLFIE) (OMIM: 605,751), an autosomal dominant disorder with incomplete penetrance, characterized by self-limited seizures occurring in infancy that respond well to medication [2]. Mutations in the *PRRT2* gene (OMIM: 614,386) have been identified as the major cause of SeLIE. As a key component of the neurotransmitter release machinery, *PRRT2* encodes proline-rich transmembrane protein 2 with 340 amino acids [3] and is involved in synaptic vesicle exocytosis and Ca^2+^ sensitivity by interacting with proteins of the fusion complex and Ca^2+^ sensors [4]. SeLIE is characterized by afebrile focal or focal to bilateral tonic-clonic seizures (FBTCS) [5]. Previous studies have shown that most antiseizure medications (ASMs) are effectively in setting seizure remission in SeLIE patients [6–10]. However, levetiracetam (LEV) is a relatively new drug, licensed in the United States in 1999 and in China in 2006, and has been reported in sporadic papers as monotherapy or in combination with other ASMs for the treatment of SeLIE [8, 9]. Therefore, the efficacy of LEV in patients with SeLIE is unclear and there is a lack of investigation to statistically analyze the efficacy. Our observations suggest that LEV may be ineffective or may even worsen the condition, something that has rarely been noticed before and the mechanism is unknown.

Therefore, we performed a retrospective study to explore the efficacy of LEV compared with other ASMs in the treatment of patients with SeLIE/ SeLFIE associated with *PRRT2* mutations.

## Materials and methods

### Individuals

The SeLIE patients with *PRRT2* mutations or 16p11.2 microdeletion including *PRRT2* were retrospectively recruited from January 2017 to June 2022 at the Department of Neurology, Guangzhou Women and Children’s Medical Center. Clinical data, including onset age of epilepsy, gender, family history, clinical manifestations, EEG, blood biochemistry, blood/urine metabolism, and brain MRI findings, were analyzed retrospectively. Based on the initial administration of ASM, the patients were divided into two groups: the levetiracetam group (LEG), and the other ASMs group (OAG). A 75-100% reduction in the frequency of seizures from baseline was considered effective [11], while an increase of more than 25% in seizure frequency from baseline was considered an exacerbation of the disease. For patients in the LEG, LEV was replaced with other ASMs in all but 3 patients due to poor efficacy in the latter stage. All patients were followed up for at least six months. Informed consent was obtained from the parents or legal guardians of the children. This study was approved by the Human Research Ethics Committee of Guangzhou Women and Children’s Medical Center.

### Genetic analysis

The peripheral blood samples of the probands and their parents were collected. Following the manufacturer’s instructions, genomic DNA was extracted from peripheral blood using the Solpure Blood DNA kit (Magen). *PRRT2* variants were identified by using next-generation sequencing of whole exome or epilepsy-associated gene panels. Sequence variants were screened and annotated using population and literature databases, including 1000 Genomes, ESP6500, dbSNP, gnomAD, HGMD, OMIM, and ClinVar. Protein structure, conserved domain and functional domain were predicted by in silico prediction tools. The interpretation of variant was manipulated in accordance with the standards and guidelines of the American College of Medical Genetics and Genomics (ACMG) [12].

### Statistical analysis

Statistical analyses were performed with SPSS18.0 (SPSS, Inc., Chicago, IL, United States). The skewness data were expressed by four-digit intervals, and the Mann-Whitney U test was used to compare the differences between the two groups. The two-tailed Fisher’s exact test was applied to compare the distribution of variants, epileptic phenotype, EEG, and gender between the two groups. The cutoff value for statistical significance was set at 0.05.

## Results

### Demographic data

A total of 39 individuals with SeLIE/SeLFIE from 38 unrelated families were included. The onset age of epilepsy ranged from 3 to 11 months, with a median age of 4 months. Thirty-eight patients had an onset age of 3 to 8 months (38/39, 97.44%; Table 1). Based on the criteria of the Commission on Classification and Terminology of the ILAE (1981, 2010, and 2017) [13–15], all patients were diagnosed with focal epilepsy, including 26 patients showing FBTCS (26/39, 66.67%), eight patients presenting with focal seizures (8/39, 20.51%), and five patients suffering from focal seizures and FBTCS (5/39, 12.82%) (Table 1). Of the 39 patients, 27 showed seizure clusters (27/39, 69.23%). Twenty-four patients from 23 families had a family history of epilepsy or paroxysmal kinesigenic dyskinesia (PKD) (24/39, 61.54%). Brain MRI was normal in all patients. No significant difference in epileptic phenotype was observed between the two groups (*χ*^*2*^ = 0.209, *P* = 1.000; Table 2).

**Table 1:** General clinical data of 39 children

Pt	Gender	Age^onset^(m)	Type of seizures	Seizures in cluster	Brain MRI	Interictal EEG	FH
1	F	8	Focal	Y	Normal	FD, unilateral, left temporal, occipital, frontal area	+
2	F	7	Focal	Y	Normal	FD, bilateral frontal areas	+
3	F	4	FBTCS	Y	Normal	MFD	+
4	M	6	FBTCS	N	Normal	Normal	-
5	M	5	FBTCS	Y	Normal	MFD + Focal-generalize	+
6	F	6	Focal	N	Normal	Normal	+
7	F	5	FBTCS	N	Normal	FD, bilateral frontal-central areas	+
8	M	11	FBTCS	Y	Normal	Normal	-
9	M	3	Focal	Y	Normal	FD, bilateral frontal-temporal areas	-
10	F	5	FBTCS	N	Normal	FD, unilateral, right, frontal	-
11	F	3	FBTCS	N	Normal	Normal	+
12	F	7	Focal	N	Normal	FD, bilateral occipital, temporal areas	-
13	M	7	FBTCS	N	Normal	Normal	+
14	F	4	FBTCS	Y	Normal	FD, bilateral occipital-temporal areas	+
15	M	4	FBTCS	Y	Normal	MFD + Focal-generalize	-
16	F	3	FBTCS	Y	Normal	MFD	+
17	M	4	FBTCS	N	Normal	FD, unilateral, right temporal areas	+
18	M	4	Focal + FBTCS	Y	Normal	FD, bilateral frontal-temporal areas	+
19	M	3	FBTCS	Y	Normal	Normal	+
20	M	3	FBTCS	Y	Normal	FD, bilateral temporal areas	-
21	M	3	FBTCS	Y	Normal	FD, bilateral central, parietal areas	+
22	M	3.5	Focal	Y	Normal	MFD, slow background activities	+
23	F	3.5	Focal	N	Normal	FD, bilateral central, parietal areas	+
24	F	5	Focal	Y	Normal	FD, bilateral occipital-temporal areas	+
25	F	4	FBTCS	N	Normal	MFD + Focal-generalize; slow background activities	-
26	F	4	FBTCS	N	Normal	FD, unilateral, left, temporal area	-
27	F	4	FBTCS	Y	Normal	MFD + Focal-generalize; slow background activities	+
28	M	4	FBTCS	N	Normal	FD, bilateral frontal, temporal areas	-
29	M	7	FBTCS	Y	Normal	FD, Slow background activities, bilaterial frontal, central areas	+
30	M	5.5	FBTCS	Y	Normal	FD, Slow background activities, bilaterial frontal, central areas	+
31	F	3	FBTCS	Y	Normal	Normal	+
32	M	5	FBTCS	Y	Normal	FD, bilateral occipital-temporal areas	-
33	M	4	FBTCS	Y	Normal	MFD; slow background activities	+
34	M	4.5	Focal + FBTCS	Y	Normal	FD, unilateral, left temporal, occipital-temporal, central area	-
35	F	5.5	Focal + FBTCS	Y	Normal	FD, bilateral occipital areas	+
36	F	5.5	Focal + FBTCS	Y	Normal	FD, bilateral frontal central areas	-
37	M	5	Focal + FBTCS	Y	Normal	FD, bilateral occipital-temporal areas	-
38	M	5	FBTCS	Y	Normal	MFD, Slow background activities,	+
39	M	6	FBTCS	Y	Normal	FD, Slow background activities, bilaterial frontal, central areas	-

**Table 2:** Clinical characteristics and therapeutic results between LEV group and Other ASMs group

	LEV group (n = 16)	Other AEDs group (n = 23)	Statistic	*P*
Gender (male/female)	10/6	11/12	*-*	0.516^#^
Onset age [*M (P*_*25*_ *~ P*_*75*_*)*, m]	5.25 (3.25 ~ 6.00)	4.00 (4.00 ~ 5.00)	*Z =* 1.174*	0.240
Distribution of variation [n (%)]				
Microdeletion	5 (31.25%)	2 (8.70%)	*χ2* = 3.542^#^	0.315
Duplication	7 (43.75%)	15 (65.22%)		
Missense	1 (6.25%)	2 (8.70%)		
Deletion	3 (18.75%)	4 (17.39%)		
EEG				
Normal	3 (18.75%)	4 (17.39%)	*χ*^*2*^ = 0.243^#^	1.000^#^
MFD	4 (25.00%)	5 (21.74%)		
FD	9 (56.25%)	14 (60.87%)		
Epileptic phenotype				
Focal	3 (18.75%)	5 (21.74%)	*χ*^*2*^ = 0.209^#^	1.000^#^
FBTCS	11 (68.75%)	15 (65.22%)		
Focal + FBTCS	2 (12.50%)	3 (13.04%)		
Therapeutic effect [n (%)]				
Effective	2 (12.50%)	22 (95.65%)	*χ*^*2*^ = 29.541^#^	0.000
Ineffective	5 (31.25%)	1 (4.35%)		
Aggravated	9 (56.25%)	0		
Effective time^∆^ [*M (P*_*25*_ *~ P*_*75*_*)*,d]	15.00 (9.50 ~ 45.00)	1.00 (1.00 ~ 1.00)	*Z* = 4.912*	0.000

*Abbreviation: FBTCS, focal to bilateral tonic-clonic seizures; FD, focal discharges; MFD, multiple focal discharges*

* Mann-Whitney U test, # Fisher’s exact test. ∆ Effective time: time from ASM treatment to seizure free

Among the 39 patients, LEV was used in 16 patients as an initial management, while other ASMs were initially adopted for the treatment of the remaining 23 patients, including 13 with valproate (VPA), seven with topiramate (TPM), and three with oxcarbazepine (OXC) (Table s2). The LEG included 16 patients (10 males and 6 females, aged 5.25 ± 2.07 months) while the OAG consisted of 23 patients (11 males and 12 females, aged 4.48 ± 1.12 months). No significant differences in gender and onset age were observed between the two groups (*P* = 0.565; *z* = 1.174, *P* = 0.240, respectively; Table 2).

### Variants

**Fig. 1:**
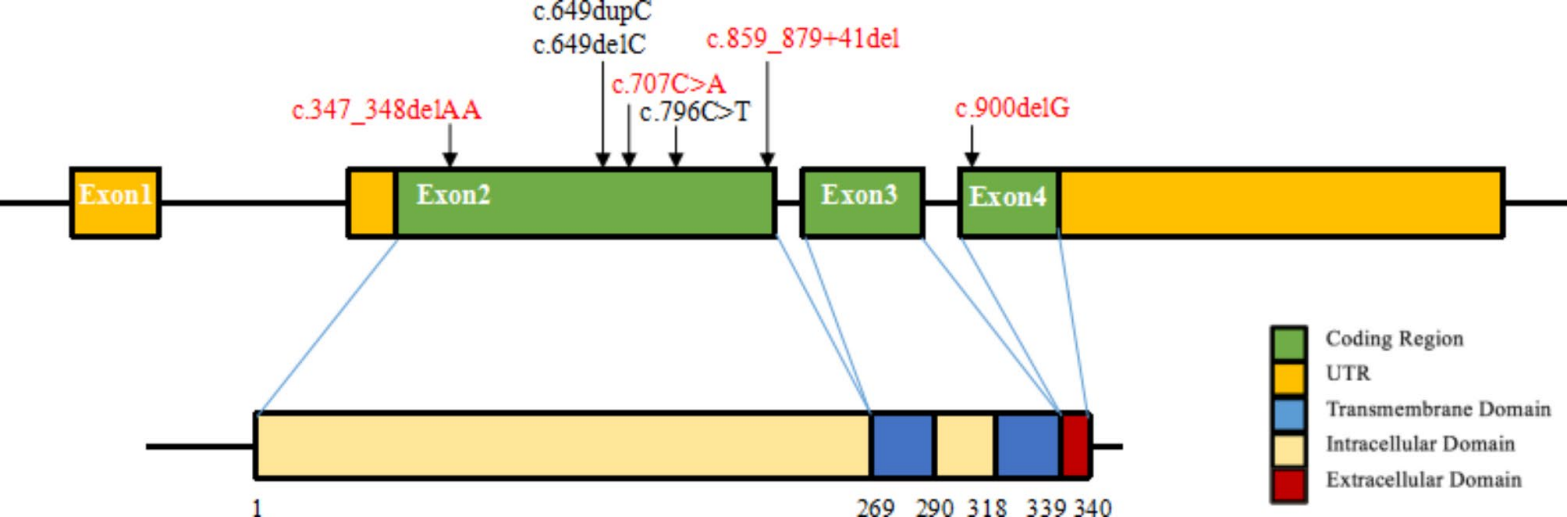
Locations of seven mutations identified in the *PRRT2* gene. Red: novel mutations identified in this study

**Fig. 2:**
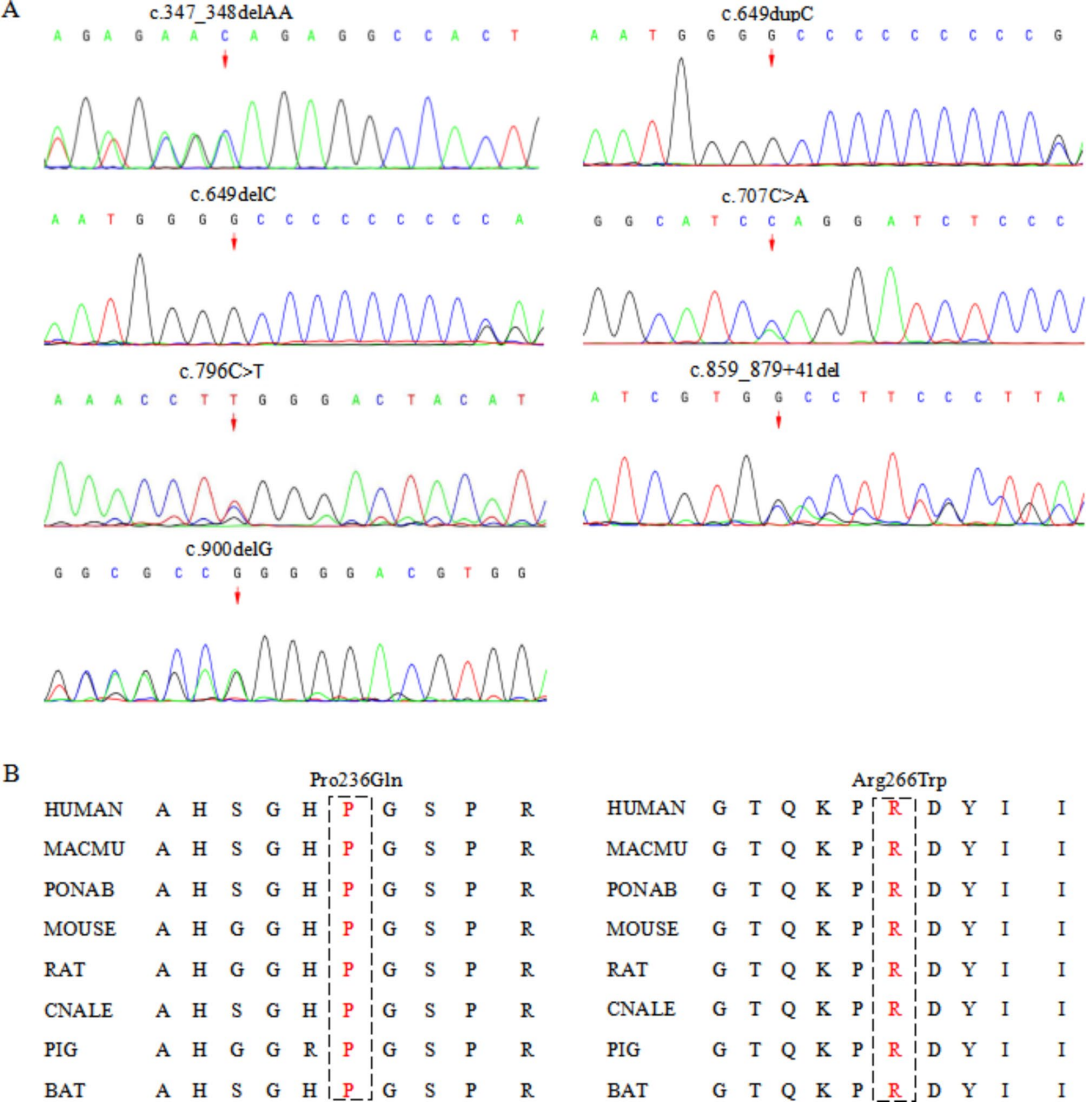
Genetic data of cases with *PRRT2* mutations. **(A)** DNA sequencing chromatograms of seven mutations. **(B)** conservative analysis of two missense mutations

Genetic analysis was performed on the 39 patients (Fig. 1, Table s1). Seven heterozygous variants were detected, including three previously reported mutations (c.649dupC, c.649delC, and c.796 C > T) and four novel variants which included one missense variant (c.707 C > A) and three small deletion variants (c.347_348delAA, c.900delG, and c.859_879 + 41del) (Fig. 2A). Seven microdeletions including *PRRT2* were also found. Among the point mutations, the hotspot mutation (c.649dupC) was detected in 22 out of 39 patients (22/39, 56.41%), and the c.649delC mutation was detected in 4 out of 39 patients (10.26%). *De novo* variants occurred in 10 individuals (10/39, 25.64%), whereas other 28 patients (28/39, 71.79%) inherited variants from their parents, and 24 out of these 28 patients (85.71%) from 27 families had a positive family history. All missense variants analyzed by in silico prediction tools were highly conserved. The prediction of c.796 C > T by SIFT, Mutation taster, Polyphen2, Condel, and M-CAP was deleterious, whereas c.707 C > A was predicted to be not deleterious by Mutation taster, Polyphen2, and Condel but deleterious by SIFT and M-CAP (Table 3). The missense variants Pro236Gln and Arg266Trp were conserved in mammals by sequence alignment (Fig. 2B). The c.859_879 + 41del variant was identified to affect the donor splice site by SpliceAI. Thus, c.707 C > A(p.Pro236Gln), c.796 C > T(p.Arg266Trp), and c.859_879 + 41del were classified as “uncertain significance,” “likely pathogenic,” and “pathogenic,” respectively, in accordance with the ACMG guidelines. Microdeletions including *PRRT2* were detected in seven patients, and six of them were 16p11.2 recurrent deletion. Among the seven patients, *de novo* microdeletions occurred in four patients (4/7, 57.1%), whereas the other two patients inherited the microdeletions from their symptomatic parents. The source of the remaining one was uncertain due to the parents’ refusal to verification.

**Table 3:** Genetics characteristics of two missense mutations identified in this study

Nucleotide alteration	Amino acid changes	gnomAD	GERP++	SIFT	Mutation Taster	Polyphen2	Condel
c.796 C > T	p.Arg266Trp	-	2.83	deleterious	disease causing (0.208)	probably_damaging (0.997)	Deleterious (0.911)
c.707 C > A	p.Pro236Gln	3.98E-06	2.95	deleterious	polymorphism (0.757)	Benign (0.103)	Neutral (0.453)

16p11.2 microdeletion, duplication, missense mutation, and deletion mutation in *PRRT2* was observed in 5 (31.25%), 7 (43.75%), 1 (6.25%), and 3 (18.75%) cases in the LEG and 2 (8.7%), 15 (65.2%), 2 (8.7%), and 4 (17.4%) cases in the OAG, respectively. No significant difference in the distribution of the variants was observed between the two groups (*χ*^*2*^ = 3.542, *P* = 0.315; Table 2).

### Electroencephalogram Data

Ictal EEG was recorded in four patients with focal seizures and FBTCS, whose synchronous EEG showed focal frontal or temporal lobe spikes, and gradually evolved to generalized spikes and slow waves. Eight patients (8/39, 20.51%) showed slow background activities. The EEG recordings from 23 patients (23/39, 58.97%) showed focal discharges, whereas those from nine patients (9/39, 23.08%) showed multifocal discharges. Normal EEG was recorded in 7 patients (7/39, 17.95%) (Table 1). No significant difference in the EEG was observed between the two groups (*χ*^*2*^ = 0.243, *P* = 1.000; Table 2).

### Pharmacological treatment

Of all patients received LEV treatment, two patients achieved a seizure-free status at 5 months old with dosage of 26.67 mg/kg/d and 21.05 mg/kg/d respectively, and one patient with regular LEV administration suffered from intermittent attacks and got relieved at 3 years old. No improvements were found in five patients with a dosage of 20–43 mg/kg/d (mean, 31.21 ± 9.86 mg/kg/d), while seizure aggravation occurred in nine patients. The dosage ranged from 16.67 mg/kg/d to 37.33 mg/kg/d (mean, 28.01 ± 7.72 mg/kg/d) in patients with seizure aggravation. In patients with seizure aggravation or no improvement, LEV was replaced with other ASMs, including VPA for seven patients, OXC for two patients, TPM for three patients, and lamotrigine (LTG) for one patient at the age of four to ten months old (nine to 93 days after LEV administration). Seizures were well controlled after LEV was replaced with other ASMs in 13 patients (table s2). In the OAG, patients who initially received VPA, OXC, and TPM obtained a seizure-free status with a dosage range of 8.89–22.22 mg/kg.day (serum concentration range from 28 to 88 µg/ml)(mean, 17.54 ± 3.26 mg/kg/d), 7.50–8.69 mg/kg/d (mean, 8.25 ± 0.65 mg/kg/d), and 0.80–2.66 mg/kg/d (median, 0.89 mg/kg/d), respectively. For those patients who continued to take ASMs, their current dosage range was 13.33 to 20.00 mg/kg/d VPA, 7.20 to 18.00 mg/kg/d OXC, 0.88 to 2.38 mg/kg/d TPM, and 1.14 mg/kg/d LTG, respectively. Only one patient with recurrent seizures who initially received TPM achieved seizure-free after replacing TPM with OXC.

Taken together, in the LEG, treatments were effective in two (12.50%), ineffective in five (31.25%), and aggravated in nine (56.25%) patients. In contrast, effective treatments were found in 22 (95.65%), ineffective in one (4.35%), and there was no aggravation in the OAG. A significant difference in effectiveness was found between the two groups (*χ*^*2*^ = 29.541, *P* = 0.000; Table 2). Currently, 21 of the 39 patients stopped taking ASMs and achieved remission.

## Discussion

*PRRT2*-related SeLIE/SeLFIE is an autosomal dominant disorder characterized by self-limited seizures that occur in infancy with an onset age of 3 months to 12 months [16]. It occurs in clusters spontaneously or in the context of other febrile disorders [17]. The pathogenic mechanism of *PRRT2* mutations remains unclear. The missense mutated *PRRT2* is clustered near the C-terminus, resulting in protein mislocalization [18]. The pathogenic mechanism may be loss of function, including haploinsufficiency or dominant negative effect. The exact pathogenic mechanisms remain to be investigated.

At present, 93 variants in *PRRT2* have been reported in the Human Gene Mutation Database, including 42 missense variants, 25 small deletions, 15 small insertions, five gross deletions, one gross insertion/duplication, and five splicing site variants. c.649dupC was the most common mutation in our series, which was consistent with previous research [4]. Recurrent 16p11.2 microdeletions were found in six patients, and whole deletion of *PRRT2* gene was found in one patient, and deletion of c.649delC was found in four patients. The identification of the novel variants, including c.859_879 + 41del, c.347_348delAA, c.707 C > A, and c.900delG, would expand the mutant spectrum of *PRRT2*.

The most common manifestation of the patients with *PRRT2* mutations is SeLIE. In rare cases, patients with *PRRT2* variants may present severe seizures [19]. Some patients may develop paroxysmal dyskinesia in adolescence or adulthood, and some have a positive family history. The affected family members may show self-limited epilepsy in childhood or paroxysmal dyskinesia in adulthood [20]. Twenty-four patients had a positive family history of epilepsy or paroxysmal kinesigenic dyskinesia in this study. All the parents who had seizures achieved remission in early childhood.

The SeLIE patients with *PRRT2* mutations usually have normal interictal EEG. In a few cases, interictal paroxysms were captured, including focal epileptiform discharges from all single regions, bilateral centrotemporal spikes [21], bilateral parietotemporal spikes [22], and unilateral frontocentral spikes [23]. Here, abnormal interictal EEG discharges were monitored in most patients. Furthermore, multifocal discharges were observed compared with previous reports.

*PRRT2*-related seizure is a type of benign epilepsy. For treatment, many ASMs such as OXC, VPA, and LEV monotherapy have been reported to achieve favorable results in these patients [8], but based on our experience, we found that LEV should not be the first option. LEV is a broad-spectrum ASMs that is effective against both focal and generalized epilepsies, and is considered to be a safe and effective treatment with a high retention rate [24]. LEV can effectively alleviate seizures in eyelid myoclonia of Jeavons syndrome [25], Dravet syndrome with mutation of *SCN1A* [26], and *PCDH19* Girls Clustering Epilepsy [27]. Here, however, our observations found LEV to be unsatisfactory as the first choice for SeLIE caused by *PRRT2* mutations. In this study, the seizure of 81.25% of patients who received LEV had aggravated seizures or ineffective treatment. Specifically, as the drug dose increased, the frequency of episodes increased and the EEG background activities deteriorated. However, in more than 90% of cases that received other ASMs in the initial choices, patients quickly got relief. Furthermore, once patients who received LEV initially and were replaced with other ASMs, remission is also achieved quickly. The single alternative drug could be VPA, OXC, LTG, or TPM, and the dose required for OXC was almost the minimum working or therapeutic dose. Although the replaced ASMs are different, seizures were well controlled within one to three days, and there was no difference in duration of effectiveness, whether another ASM was preferred as the first choice or as an alternative for ineffective treatment of LEV. Therefore, LEV may be an unsuitable medication for patients with SeLIE caused by *PRRT2* mutations. For focal or focal origin seizures, OXC is a worthy priority for *PRRT2*-mutated patients, which is also effective for possible PKD in teen years [28]. It has recently been reported that VPA showed poor results on *PRRT2*-related epilepsy [29], and only three patients were treated with VPA. In this study, all the twenty patients who took VPA obtained seizure-free status, of which 13 patients initially took VPA in OAG and 7 patients switched to VPA in LEG. It is indicated that VPA has a good therapeutic effect on *PRRT2-*related epilepsy. Nevertheless, given the retrospective nature of our study and the limited sample size, more cases at multiple epilepsy centers will help validate our findings in the future.

A few studies on LEV’s ineffectiveness have been reported in patients with SeLIE associated with *PRRT2* mutations [29, 30], and the mechanism of LEV’s inefficacy or exacerbation of *PRRT2*-related epilepsy is unclear. The synaptic vesicle protein 2 A (SV2A) is identified to be the binding site for LEV in the central nervous system, and it is a membrane protein present on all synaptic vesicles that can stabilize and transport the calcium-sensor protein synaptotagmin1 [31]. *PRRT2* is localized to axons, and is associated with glutamatergic synapses [32]. It interacts with the synaptic proteins, namely SNAP-25, VAMP2 and synaptotagmin1/2 [33]. Loss-of-function mutations in *PRRT2* might lead to altered synaptic vesicle release. Downregulation of *PRRT2* affects the Ca^2+^ coupling between action potential and exocytosis by decreasing the probability of release pulse ratio, thereby rendering facilitation more intense in excitatory synapses or depression milder in inhibitory synapses [33], which indicated that *PRRT2* is closely connected with the Ca^2+^-sensing machinery and that it plays an important role in the final steps of neurotransmitter release and affects neuronal excitability. We hypothesized that *PRRT2* mutation might change the binding of synaptotagmin1; thus, the interaction between synaptotagmin1 and SV2A protein may be changed. However, the effect of LEV combined with SV2A may interfere with neurotransmitter release, and the mechanisms should be further investigated.

It has been reported that patients with SeLFIE have a natural remission by the age of 3 [34]. Some scholars suggest that ASMs can probably be withdrawn after 1 to 2 years of seizure freedom [35]. In our study, all patients obtained seizure control at their last visit between 4 months and 3 years of age. Cluster seizures recurred in one patient who was stopped to take VPA by her family at the age of 1 year and a half, and the seizure was re-controlled after the re-application of VPA, indicating that the patient was not in natural remission at that time. Another child who received LEV suffered from persistent seizures until age 3, combined with the withdrawal of ASMs in 21 other children, indicating that it is safe to stop ASMs treatment at age 3.

## Conclusion

*PRRT2*-related epilepsy is a self-limited epilepsy that may be accompanied by various EEG abnormalities. In this study, most SeLIE patients caused by *PRRT2* mutations benefited more from most ASMs than LEV, and neurologists should be cautious in selecting LEV as the initial ASM for these patients.

### Electronic supplementary material

Below is the link to the electronic supplementary material.

Supplementary Material 1

Supplementary Material 2

## Data Availability

The raw data supporting the conclusions of this article will be made available by the authors, without undue reservation.

## Abbreviations

SeLIE: self-limited infantile epilepsy
ASM: Antiseizure medication
FBTCS: focal to bilateral tonic-clonic seizures
LEG: levetiracetam group
OAG: other ASMs group
PKD: Paroxysmal kinesigenic dyskinesia
PRRT2: proline-rich transmembrane protein 2
SV2A: synaptic vesicle protein 2 A
SeLFIE: self-limited familial infantile epilepsy
