# Supplementary material for: Levetiracetam may be an unsuitable choice for patients with PRRT2-associated self-limited infantile epilepsy
Source: BMC Pediatr. 2023 Oct 25;23:529. doi: 10.1186/s12887-023-04212-w (PMC10601096; doi:10.1186/s12887-023-04212-w)
Supplement: Supplementary file 1 — Supplementary Material 1 [file 12887_2023_4212_MOESM1_ESM.docx]

**Table s1 Genetic variation in 39 children**

| Pt  ID | nucleotide  Alteration | **Amino acid changes** | **Position**  **Chr 16** | **Mutation**  **type** | **Original** |  | **ACMG score** | **ACMG pathogenicity** |
| --- | --- | --- | --- | --- | --- | --- | --- | --- |
| 1 | c.649dupC | p.Arg217ProfsTer8 | 29,825,015 | Frameshift | paternal# | reported | PVS1+PS1+PM1+PP1+PP4 | P |
| 2 | c.649dupC | p.Arg217ProfsTer8 | 29,825,015 | Frameshift | maternal* | reported | PVS1+PS1+PM1+PP1 | P |
| 3 | c.649dupC | p.Arg217ProfsTer8 | 29,825,015 | Frameshift | maternal* | reported | PVS1+PS1+PM1+PP1 | P |
| 4 | c.649dupC | p.Arg217ProfsTer8 | 29,825,015 | Frameshift | *de novo* | reported | PVS1+PS1+PM1+PP1 | P |
| 5 | c.347_348del | p.Lys116ArgfsTer17 | 29,824,720 | Frameshift | maternal# | novel | PVS1+PM2+PP1 | P |
| 6 | c.796C>T | p.Arg266Trp | 29,825,171 | Missense | maternal | reported | PS1+PM2+PP3+PP4 | LP |
| 7 | c.796C>T | p.Arg266Trp | 29,825,171 | Missense | maternal | reported | PS1+PM2+PP3+PP4 | LP |
| 8 | c.707C>A | p.Pro236Gln | 29,825,082 | Missense | maternal | novel | PM2+PP3+PP4 | VUS |
| 9 | c.649dupC | p.Arg217ProfsTer8 | 29,825,015 | Frameshift | *de novo* | reported | PVS1+PS1+PS2+PM1+PP4 | P |
| 10 | c.649dupC | p.Arg217ProfsTer8 | 29,825,015 | Frameshift | de novo | reported | PVS1+PS1+PM1+PP1+PP4 | P |
| 11 | c.649dupC | p.Arg217ProfsTer8 | 29,825,015 | Frameshift | paternal* | reported | PVS1+PS1+PM1+PP1+PP4 | P |
| 12 | c.649dupC | p.Arg217ProfsTer8 | 29,825,015 | Frameshift | *de novo* | reported | PVS1+PS1+PS2+PM1+PP4 | P |
| 13 | c.649dupC | p.Arg217ProfsTer8 | 29,825,015 | Frameshift | maternal* | reported | PVS1+PS1+PM1+PP1+PP4 | P |
| 14 | c.649dupC | p.Arg217ProfsTer8 | 29,825,015 | Frameshift | paternal* | reported | PVS1+PS1+PM1+PP1+PP4 | P |
| 15 | c.649dupC | p.Arg217ProfsTer8 | 29,825,015 | Frameshift | maternal | reported | PVS1+PS1+PM1+PP4 | P |
| 16 | c.649dupC | p.Arg217ProfsTer8 | 29,825,015 | Frameshift | maternal* | reported | PVS1+PS1+PP1+PP4 | P |
| 17 | c.649delC | p.Arg217GlufsTer12 | 29,825,016 | Frameshift | maternal | reported | PVS1+PS1+PM1+PP4 | P |
| 18 | c.649delC | p.Arg217GlufsTer12 | 29,825,016 | Frameshift | paternal | reported | PVS1+PS1+PS2+PM1+PP4 | P |
| 19 | c.649delC | p.Arg217GlufsTer12 | 29,825,016 | Frameshift | paternal* | reported | PVS1+PS1+PM1+PP1+PP4 | P |
| 20 | c.649delC | p.Arg217GlufsTer12 | 29,825,016 | Frameshift | *de novo* | reported | PVS1+PS1+PS2+PM1+PP4 | P |
| 21 | c.859_879+41del | - | 29,825,234 | Frameshift | paternal* | novel | PVS1+PM2+PP1+PP3+PP4 | P |
| 22 | c.649dupC | p.Arg217ProfsTer8 | 29,825,015 | Frameshift | maternal# | reported | PVS1+PS1+PM1+PP1+PP4 | P |
| 23 | c.649dupC | p.Arg217ProfsTer8 | 29,825,015 | Frameshift | paternal*# | reported | PVS1+PS1+PM1+PP1+PP4 | P |
| 24 | c.649dupC | p.Arg217ProfsTer8 | 29,825,015 | Frameshift | maternal* | reported | PVS1+PS1+PM1+PP1+PP4 | P |
| 25 | c.900delG | p.Asp302ThrfsTer11 | 29,825,673 | Frameshift | maternal | novel | PVS1+PM2+PP4 | P |
| 26 | c.649dupC | p.Arg217ProfsTer8 | 29,825,015 | Frameshift | *de novo* | reported | PVS1+PS1+PM1+PP4 | P |
| 27 | c.649dupC | p.Arg217ProfsTer8 | 29,825,015 | Frameshift | maternal* | reported | PVS1+PS1+PM1+PP1+PP4 | P |
| 28 | c.649dupC | p.Arg217ProfsTer8 | 29,825,015 | Frameshift | maternal | reported | PVS1+PS1+PM1+PP4 | P |
| 29 | c.649dupC | p.Arg217ProfsTer8 | 29,825,015 | Frameshift | maternal* | reported | PVS1+PS1+PM1+PP1+PP4 | P |
| 30 | c.649dupC | p.Arg217ProfsTer8 | 29,825,015 | Frameshift | paternal | reported | PVS1+PS1+PM1+PP1+PP4 | P |
| 31 | c.649dupC | p.Arg217ProfsTer8 | 29,825,015 | Frameshift | paternal | reported | PVS1+PS1+PM1+PP1+PP4 | P |
| 32 | c.649dupC | p.Arg217ProfsTer8 | 29,825,015 | Frameshift | paternal | reported | PVS1+PS1+PM1+PP1+PP4 | P |
| 33 | / | / | 29,674,991-30,199,601 | Deletion | maternal* | / | / | / |
| 34 | / | / | whole gene | Deletion | *de novo* | / | / | / |
| 35 | / | / | 29,675,049-30,199,897 | Deletion | paternal* | / | / | / |
| 36 | / | / | 29,802,081-30,200,285 | Deletion | de novo | / | / | / |
| 37 | / | / | 29674991-  30199455 | Deletion | de novo | / | / | / |
| 38 | / | / | 29,458,122-30,366,038 | Deletion | / | / | / | / |
| 39 | / | / | 29,674,991-30,199,601 | Deletion | de novo | / | / | / |

*Abbreviation: * had seizures or convulsion during childhood, # showed paroxysmal kinesigenic dyskinesia*
