# Supplementary material for: Levetiracetam may be an unsuitable choice for patients with PRRT2-associated self-limited infantile epilepsy
Source: BMC Pediatr. 2023 Oct 25;23:529. doi: 10.1186/s12887-023-04212-w (PMC10601096; doi:10.1186/s12887-023-04212-w)
Supplement: Supplementary file 2 — Supplementary Material 2 [file 12887_2023_4212_MOESM2_ESM.docx]

**Table s2 Administration of ASM in 39 patients**

| Pt ID | Age^onset^ (m) | ASM^i^ | ASM dosage^i^ (mg/kg/d) | LEV dosage^f^ (mg/kg/d) | Age^r^ (m) | ASM^r^ | Dosage^w^ (mg/kg/d) | SC  (ug/ml) | ASM^c^ | Dosage^c^  (mg/kg/d) | Age^sc^ | Age^dc^ |
| --- | --- | --- | --- | --- | --- | --- | --- | --- | --- | --- | --- | --- |
| 1 | 7 | LEV | 15.38 | 37.33 | 10 | VPA | 19.20 | 54 | ceased | / | 10m | 3y2m |
| 2 | 6 | LEV | 14.28 | 34.28 | 7 | LTG | 0.31 | / | LTG | 1.14 | 8m | / |
| 3 | 4 | LEV | 17.02 | 29.79 | 5 | TPM | 0.88 | / | TPM | 0.88 | 5m | / |
| 4 | 6 | LEV | 11.11 | 22.22 | 7 | VPA | 17.78 | 67 | ceased | / | 7m | 3y |
| 5 | 5 | LEV | 11.76 | 20.00 | 5 | OXC | 9.88 | / | OXC | 9.03 | 6m | / |
| 6 | 6 | VPA | 16.00 | / | / | / | 16.00 | 44 | ceased | / | 11m | 3y1m |
| 7 | 5 | VPA | 20.00 | / | / | / | 22.22 | 61 | ceased | / | 9m | 5y |
| 8 | 11 | LEV | 20.00 | 43.00 | / | / | / | / | ceased | / | 3y | 3y |
| 9 | 3 | LEV | 10.00 | 37.50 | 4 | VPA | 15.00 | 49 | ceased | / | 4m | 3y2m |
| 10 | 5 | TPM | 0.89 | / | / | / | 1.92 | / | TPM | 1.66 | 6m | / |
| 11 | 3 | LEV | 16.67 | 33.33 | 4 | VPA | 20.00 | 38 | ceased | / | 5m | 3y |
| 12 | 7 | VPA | 20.00 | / | / | / | 20.00 | 50 | ceased | / | 9m | 3y5m |
| 13 | 7 | VPA | 8.89 | / | / | / | 8.89 | NA | ceased | / | 8m | 3y1m |
| 14 | 4 | VPA | 17.45 | / | / | / | 19.20 | 65 | ceased | / | 4.5m | 2y11m |
| 15 | 4 | TPM | 1.00 | / | / | / | 1.00 | / | ceased | / | 5m | 3y |
| 16 | 3 | VPA | 17.14 | / | / | / | 17.14 | 60 | ceased | / | 5m | 3y |
| 17 | 4 | OXC | 8.57 | / | / | / | 8.57 | / | ceased | / | 5m | 4y |
| 18 | 4 | OXC | 7.50 | / | / | / | 7.50 | / | OXC | 12.00 | / | / |
| 19 | 3 | VPA | 19.20 | / | / | / | 19.20 | 36 | VPA | 17.78 | 4m | / |
| 20 | 3 | LEV | 9.00 | 36.14 | 7 | VPA | 19.20 | 63 | VPA | 17.39 | 7m | / |
| 21 | 5 | LEV | 11.32 | 18.87 | 6 | VPA | 18.90 | 28 | VPA | 13.33 | 6m | / |
| 22 | 3 | TPM | 0.89 | / | / | / | 0.89 | / | ceased | / | 4m | 3y |
| 23 | 3 | OXC | 8.69 | / | / | / | 8.69 | / | OXC | 7.20 | 4m | / |
| 24 | 5 | VPA | 16.00 | / | / | / | 16.67 | 88 | ceased | / | 5m | 2y6m |
| 25 | 4 | TPM | 0.80 | / | / | / | 0.80 | / | ceased | / | 4m | 2y7m |
| 26 | 4 | VPA | 15.58 | / | / | / | 15.58 | 70 | ceased | / | 4m | 3y |
| 27 | 4 | VPA | 20.00 | / | / | / | 20.00 | 61 | ceased | / | 7m | 3y |
| 28 | 4 | VPA | 18.60 | / | / | / | 18.60 | 40 | ceased | / | 6m | 3y |
| 29 | 7 | LEV | 16.67 | 16.67 | 7 | TPM | 0.52 | / | TPM | 1.33 | 1y4m | / |
| 30 | 5 | VPA | 20.00 | / | / | / | 20.00 | 38 | VPA | 20.00 | 6m | / |
| 31 | 3 | TPM | 2.66 | / | 4 | OXC | 10.00 | / | OXC | 10.00 | 4m | / |
| 32 | 5 | TPM | 1.38 | / | / | / | 1.38 | / | TPM | 2.27 | 5m | / |
| 33 | 4 | LEV | 10.00 | 26.67 | / | / | 20.00 | / | LEV | 20.00 | 5m | / |
| 34 | 4 | VPA | 17.00 | / | / | / | 17.00 | 50 | ceased | / | 5m | 2y5m |
| 35 | 5 | LEV | 19.35 | 29.03 | 5 | VPA | 19.35 | 28 | ceased | / | 6m | 3y |
| 36 | 5 | LEV | 17.50 | 30.00 | 6 | OXC | 15.00 | / | OXC | 18.00 | 1y2m | / |
| 37 | 5 | TPM | 0.83 | / | / | / | 0.83 | / | TPM | 1.47 | 5m | / |
| 38 | 5 | LEV | 12.50 | 21.05 | / | / | 21.05 | / | LEV | 18.18 | 5m | / |
| 39 | 6 | LEV | 14.29 | 20.00 | 7 | TPM | 1.79 | / | TPM | 2.38 | 6.5m | / |

*Abbreviation: age^onset^, onset age; ASM^i^, initial ASM; ASM dosage^i^, initial ASM dosage; LEV dosage^f^, final LEV dosage; Age^r^, Age replaced ASM; ASM^r^, replaced ASM; dosage^w^, working dosage; SC, serum concentration; ASM^c^, current ASM; dosage^c^, current dosage; Age^sc^, seizure control age; Age^dc^, ASM ceasing age.*
